# Nicotinic Acid Adenine Dinucleotide Phosphate (NAADP) and Cyclic ADP-Ribose (cADPR) Mediate Ca^2+^ Signaling in Cardiac Hypertrophy Induced by β-Adrenergic Stimulation

**DOI:** 10.1371/journal.pone.0149125

**Published:** 2016-03-09

**Authors:** Rukhsana Gul, Dae-Ryoung Park, Asif Iqbal Shawl, Soo-Yeul Im, Tae-Sik Nam, Sun-Hwa Lee, Jae-Ki Ko, Kyu Yoon Jang, Donghee Kim, Uh-Hyun Kim

**Affiliations:** 1 Department of Biochemistry, Chonbuk National University Medical School, Jeonju, Korea; 2 National Creative Research Laboratory for Ca^2+^ signaling Network, Chonbuk National University Medical School, Jeonju, Korea; 3 Department of Internal Medicine, Chonbuk National University Medical School, Jeonju, Korea; 4 Department of Pathology, Chonbuk National University Medical School, Jeonju, Korea; 5 Institute of Cardiovascular Research, Chonbuk National University Medical School, Jeonju, Korea; 6 Department of Physiology and Biophysics, Chicago Medical School, Rosalind Franklin University of Medicine and Science, North Chicago, Illinois, United States of America; University of Western Ontario, CANADA

## Abstract

Ca^2+^ signaling plays a fundamental role in cardiac hypertrophic remodeling, but the underlying mechanisms remain poorly understood. We investigated the role of Ca^2+^-mobilizing second messengers, NAADP and cADPR, in the cardiac hypertrophy induced by β-adrenergic stimulation by isoproterenol. Isoproterenol induced an initial Ca^2+^ transients followed by sustained Ca^2+^ rises. Inhibition of the cADPR pathway with 8-Br-cADPR abolished only the sustained Ca^2+^ increase, whereas inhibition of the NAADP pathway with bafilomycin-A1 abolished both rapid and sustained phases of the isoproterenol-mediated signal, indicating that the Ca^2+^ signal is mediated by a sequential action of NAADP and cADPR. The sequential production of NAADP and cADPR was confirmed biochemically. The isoproterenol-mediated Ca^2+^ increase and cADPR production, but not NAADP production, were markedly reduced in cardiomyocytes obtained from CD38 knockout mice. CD38 knockout mice were rescued from chronic isoproterenol infusion-induced myocardial hypertrophy, interstitial fibrosis, and decrease in fractional shortening and ejection fraction. Thus, our findings indicate that β-adrenergic stimulation contributes to the development of maladaptive cardiac hypertrophy via Ca^2+^ signaling mediated by NAADP-synthesizing enzyme and CD38 that produce NAADP and cADPR, respectively.

## Introduction

In cardiac cells, Ca^2+^ has a fundamental role in the regulation of both contraction and transcription [1–3]. Cardiac hypertrophy and heart failure may arise from inappropriate remodeling of the Ca^2+^ signaling components [4]. The heart is a highly plastic organ that undergoes hypertrophy in response to different types of stimuli [5,6]. The remodeling responsible for cardiac hypertrophy is a compensatory mechanism that increases the stroke volume to meet the work demand of the body, and it is reversible, in that the heart will return to its original size if the abnormal stimuli are removed within a critical time period [7,8] If certain pathological conditions persist, however, the cardiac hypertrophy can become decompensated, shifting to the more irreversible state of heart failure [9,10]. It is therefore important to determine which Ca^2+^ signaling processes control the development of cardiac hypertrophy.

Persistent activation of the β-adrenergic and renin-angiotensin-aldosterone systems (RAS) is a hallmark of cardiac hypertrophy and subsequent heart failure, in which norepinephrine (NE) and angiotensin II (AngII) are the primary effectors that mediate the hypertrophic and fibrotic events in the heart [11–14]. Therefore, most current therapies for heart failure are focused on antagonizing the adrenergic system and RAS [15]. NE and AngII bind to their respective G-protein-coupled receptors (GPCRs) and induce several intracellular signaling pathways involved in the hypertrophic remodeling of the heart. Studies in animal and human models show that abnormalities in Ca^2+^ handling is an important cause of hypertrophy and failing of the myocardium that occur in response to a long term and sustained stimulation by NE or AngII [16]. Thus, a clear understanding of the mechanisms by which NE and AngII modulate Ca^2+^ signaling in cardiomyocytes is important.

Mounting evidence indicates that cyclic ADP-ribose (cADPR) and nicotinic acid adenine dinucleotide phosphate (NAADP) play an essential role in Ca^2+^ mobilization from Ca^2+^ stores such as during excitation-contraction coupling in the heart [17–20]. ADP-ribosyl cyclases (ARCs) produce two NAD(P)-derived Ca^2+^ mobilizing second messengers, cADPR and NAADP [18,21]. One ARC named CD38 has been cloned and characterized, and is expressed ubiquitously [22]. However, there are as-yet-unidentified non-CD38 ARCs and NAADP-synthesizing enzymes in mammalian tissues, including the heart [23–26]. Our recent study showed that the AngII-induced increase in [Ca^2+^]_i_ was not significantly different in cardiomyocytes obtained from CD38 knockout (KO) and wild-type (WT) mice, and was completely inhibited by 8-bromo-cADP-ribose (8-Br-cADPR), indicating that cADPR was generated by an ARC other than CD38 [23]. We have recently demonstrated that bisphenyl compounds, 4,4’- and 2,2’-dihydroxyazobenzene (4DAB or 2DAB), inhibit the activity of non-CD38 ARCs in murine mesangial cells and cardiomyocytes, respectively [27,28]. Moreover, these compounds inhibited AngII-mediated cADPR formation and hypertrophic responses in the kidney and heart [27,29]. Taken together, these data indicate that aberrant expression or abnormal activity of ARCs results in pathological cardiac hypertrophy due to altered Ca^2+^ signaling and suggest that components of Ca^2+^ signaling systems could be potential targets for pharmaceutical intervention.

The aim of the present study was to identify the roles of CD38 and non-CD38 ARCs and the signals generated by these enzymes in Ca^2+^ signaling during cardiac hypertrophic remodeling. We focused on Ca^2+^ signaling in a well-established β-adrenergic receptor (β-AR) signaling pathway that produces cardiac hypertrophy.

## Materials and Methods

### Animals

Sprague-Dawley male rats were obtained from Orientbio Inc. (Seoungnam, Korea). CD38 knockout mice (CD38 KO; B6.129P2-Cd38^tm/Lud^) and CD38 wild type mice (CD38 WT) were purchased from The Jackson Laboratory (Bar Harbor, ME). Animals were housed in a 12 hour light-dark schedule with food and water ad libitum. All studies conformed to the Guide for the Care and Use of Laboratory Animals published by the US National Institutes of Health (NIH Publication No. 85–23, revised 1996). The entire project was reviewed and approved by the Institutional Animal Care and Use Committee of the Chonbuk National University Medical School (CBU 2014–00051).

### Osmotic Mini pump Infusion of Isoproterenol

To generate β-adrenergic-induced cardiac hypertrophy, isoproterenol (ISO, Sigma-Aldrich) (10 mg/kg/day) or vehicle (ddH_2_O) was administrated to 8-week-old male CD38 KO and WT mice via osmotic mini-pumps (Alzet) implanted subcutaneously. Hearts were isolated and analyzed for cardiac hypertrophy following 7 days of ISO infusion.

### Echocardiography

Left ventricular (LV) dimensions were assessed by echocardiography using a GE Vivid 4 ultrasound machine (GE Medical Systems, Waukesha, Wisconsin, USA) equipped with a 13-MHz phase array linear transducer. M-mode images were used for measurement of wall thickness, chamber dimension, fractional shortening (FS), and ejection fraction (EF) in anesthetized rats. EF provides a global assessment of left ventricular systolic performance and can be estimated using the equation described previously [27].

### Histological Analysis

Hearts were fixed with 4% paraformaldehyde and embedded in paraffin, and 6-μm thick sections were stained with hematoxylin and eosin or Masson’s trichrome [27]. Images were acquired using a Nikon ECLIPSE E600 microscope (Nikon Japann) with a 40x objective lens (Plan Apo40/0.95, Nikon, Japan) and a digital camera (Nikon DXm1200F, Nikon, Japan) and software (Nikon ACT-12.62, Nikon, Japan). The area of one image was 0.086 mm^2^. The number of cardiomyocytes per high-powered field (× 400) was counted by two pathologists. To evaluate the fibrosis of the heart, four images were taken per group at 400x magnification with Masson’s Trichrome staining (MTS) slides. The images were analyzed with image analyzing software (analySIS, Soft Imaging Systems, Germany), and the MTS-positive area was calculated as following: MTS-positive area = (MTS-stained blue-colored area/total area) x 100 (%).

### Preparation of Cardiomyocytes

Ventricular cardiomyocytes were isolated from rats (200–220 g) and C57BL/6 CD38 KO and WT mice (25–30 g) using a modification of the method described previously [23]. Briefly, animals were sacrificed by cervical dislocation. Isolated cardiomyocytes were seeded on plates previously coated with laminin (10 μg/ml; Sigma) for 1 hour. After incubation for 20 minutes at 37°C in a humidified incubator with 5% CO_2_/95% air atmosphere, the medium was changed to remove round and unattached cells. This technique routinely yields > 90% cardiomyocytes that retain a rod-shaped morphology. Experiments were performed 12–16 hours after isolation. Before treatment with blockers or ISO, cardiomyocytes were incubated in a buffer containing 20 mM HEPES, 137 mM NaCl, 4.9 mM KCl, 1.2 mM MgSO_4_, 15 mM glucose, and 1 mM Ca^2+^ for 20 minutes.

### Pretreatment of Cardiomyocytes

Cardiomyocytes were treated with the indicated concentrations of blockers and incubated at 37°C for 30 minutes prior to ISO-treatment as follows: H89 (10 μM), Rp-cAMP (100 μM), thapsigargin (10 μM), 8-Br-cADPR (100 μM), DAB (1 μM), xestospongin C (2 μM), nifedipine (10 μM), SKF 96365 (10 μM) and bafilomycin A1 (1 μM).

### [Ca^2+^]_i_ Measurement

Cardiomyocytes attached to laminin-coated dishes were loaded with the Ca^2+^ indicator fluo 3-AM (3 μM) (Molecular Probes/Life Technologies, Grand Island, NY) and incubated for 20 minutes at 37°C. Changes in [Ca^2+^]_i_ were determined at 488 nm excitation/530 nm emission by an air-cooled argon laser system [23]. The emitted fluorescence was collected using a photomultiplier. One image was scanned every 3 s using a confocal microscope (Nikon). [Ca^2+^]_i_ calculation was performed using the equation [Ca^2+^]_i_ = K_d_ (F − F_min_)/(F_max_ − F), where the dissociation constant (K_d_) is 450 nM for fluo3 and F is the observed fluorescence levels [30]. Each tracing was calibrated for the maximal intensity (F_max_) by the addition of 8 μM ionomycin and for the minimal intensity (F_min_) by the addition of 50 mM EGTA at the end of each measurement.

### Measurement of Intracellular cADPR and NAADP Concentrations

A cyclic enzymatic assay was used to measure cADPR and NAADP levels as described previously [23]. Fluorescence was measured at excitation/emission wavelengths of 544/590 nm using a fluorescence plate reader (Molecular Devices Corp., Spectra-Max GEMINI).

### Immunoblotting

Protein extraction and immunoblotting of cardiomyocytes were performed as previously described [23,27]. Signals were detected using the enhanced chemiluminescence system (Bio-Rad, Munich, Germany). For quantitative measurements, blots were scanned, and band density was normalized to the density of a control protein band for each sample. Protein concentrations were determined using the Bio-Rad protein assay kit, and known concentrations of BSA were used as the standard.

### Lentiviral Transduction

Isolated rat cardiomyocytes were plated in 6-well plates 8 hours prior to viral infection. Cardiomyocytes were transduced with lentiviral particles encoding CD38 from Santa Cruz Biotechnology (Santa Cruz, CA) at a multiplicity of infection (MOI) of 1–3. Lentiviral transduction was performed essentially according to the manufacturer's protocol. Briefly, the media was replaced with media 199 (Invitrogen, Grand Island, NY) containing 5 μg/ml polybrene. Cells were infected overnight with lentiviral particles containing CD38 or control shRNA. The next day, the media was changed and cells were incubated for 12 hours before analysis. Knockdown efficiency was confirmed by immunoblotting.

### Knockdown of Stim1 by small interference RNA (siRNA)

Stim1 siRNA was purchased from Invitrogen (Cat. 64647, 151019). Cardiac cells were cultured in an antibiotic-free growth media supplemented with fetal bovine serum. After 6 hours, cardiac cells were transfected with 60 pmol of siRNA oligonucleotides specific to Stim1 using transfection reagent (Quagen. cat.301305) without antibiotics according to the instructions of the manufacturer. After 48 hours of transfection, cells were prepared for examination.

### Statistical Analysis

Data represent means ± standard error of the mean (SEM) of at least three separate experiments. Differences were compared by analysis of variance (ANOVA) followed by Student’s t-tests. Two-group analysis was performed using Student’s t-test (paired or unpaired, as appropriate). Statistical analysis for the MTS-positive area was performed by one-way ANOVA followed by a Tukey HSD post-hoc test. A value of P < 0.05 was considered significant.

## Results

### Isoproterenol Induces a Sustained [Ca^2+^]_i_ Rise with Sequential Generation of NAADP and cADPR in Cardiomyocytes

**Fig 1:**
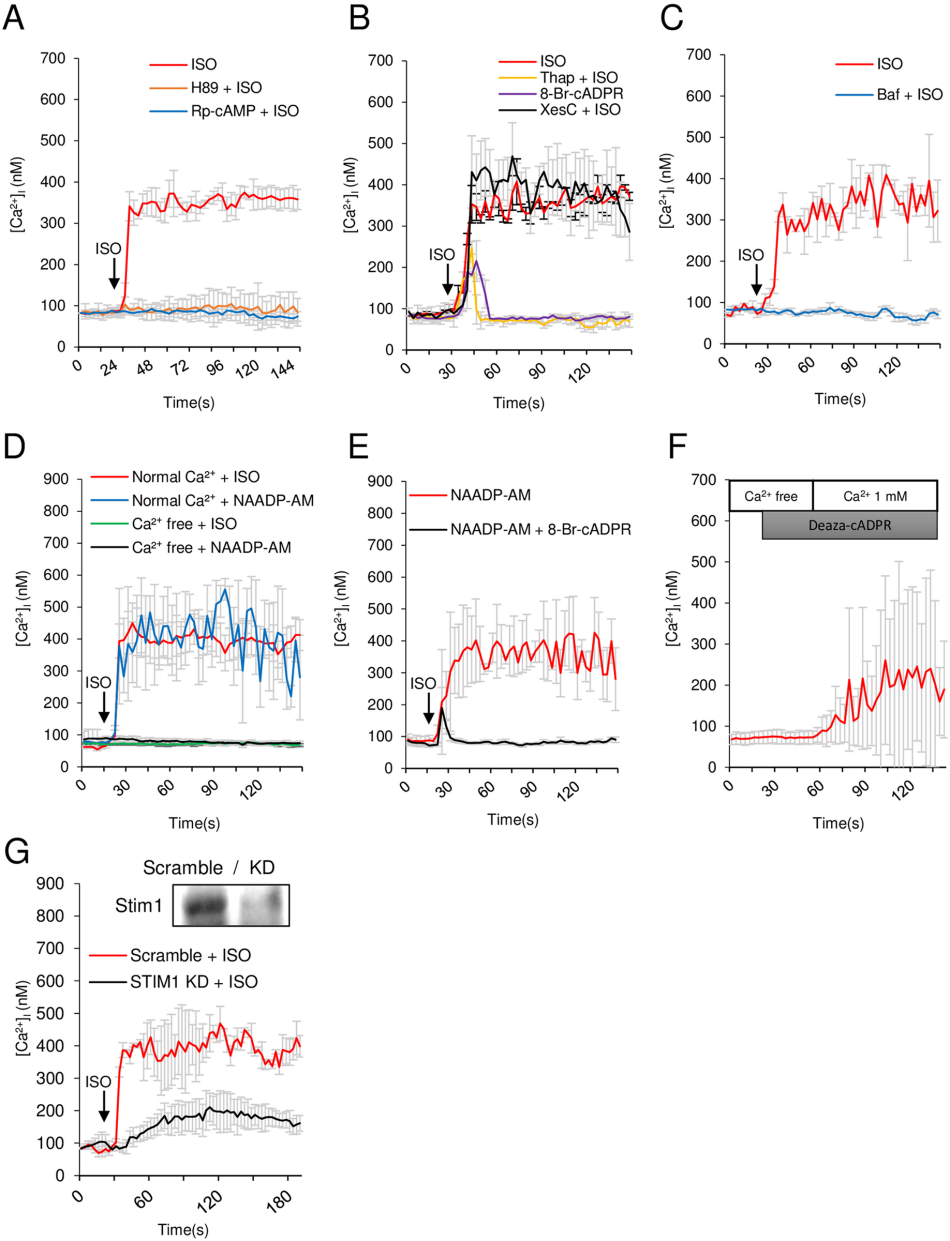
ISO-stimulated Ca^2+^ increase in cardiomyocytes. **(A)** Representative tracings of the [Ca^2+^]_i_ response to 2 μM ISO in the presence of an adenylate cyclase inhibitor and a cAMP antagonist. **(B, C)** Representative tracings of the [Ca^2+^]_i_ response to 2 μM ISO in the presence of thapsigargin (Thap), 8-Br-cADPR, xestospongine C (XesC), or bafilomycin A1 (Baf). **(D)** Representative tracings of the [Ca^2+^]_i_ response to 2 μM ISO or 50 nM NAADP-AM in the presence or absence of extracellular Ca^2+^. **(E)** Representative tracings of the [Ca^2+^]_i_ response to NAADP-AM after pretreatment with or without 8-Br-cADPR. **(F)** Representative tracings of the [Ca^2+^]_i_ response to 100 nM deaza-cADPR in the absence and presence of extracellular Ca^2+^. **(G)** Representative tracings of the [Ca^2+^]_i_ response to 2 μM ISO in cardiomyocytes after transfection with scrambled or Stim1 siRNA. Values are the mean ± SEM of three independent experiments.

Treatment of cardiomyocytes with ISO (2 μM) induced a rapid increase in [Ca^2+^]_i_ followed by a sustained increase (Fig 1A). Pretreatment of cardiomyocytes with a protein kinase A (PKA) inhibitor (10 μM H89 or Rp-cAMP) abolished the ISO-induced Ca^2+^ signaling (Fig 1A), indicating that cAMP was responsible for the ISO–induced Ca^2+^ signaling. Thapsigargin, a sarcoplasmic/endoplasmic reticulum Ca^2+^ ATPase (SERCA) inhibitor, blocked the sustained phase of the ISO–induced Ca^2+^ signal, but not the initial spike (Fig 1B). Similarly, pretreatment of cells with 8-Br-cADPR, a competitive inhibitor of cADPR, resulted in an inhibition of the sustained phase of the Ca^2+^ signal, but not the initial transient signal (Fig 1B). By contrast, xestospongin C (XesC), an IP_3_ receptor blocker, had no effect on the ISO–induced Ca^2+^ signal (Fig 1B), indicating that ISO–induced sustained Ca^2+^ signal did not require activation of IP_3_ receptors.

NAADP acts on acidic Ca^2+^ storage organelles to induce Ca^2+^ mobilization in a variety of cell types [31–33]. Bafilomycin A1, an inhibitor of vacuolar H^+^-ATPase, is known to block the Ca^2+^ release elicited by NAADP [24,31]. To determine whether the thapsigargin-insensitive initial Ca^2+^ transient was due to Ca^2+^ release from acidic Ca^2+^ stores, we tested the effect of bafilomycin A1 on ISO-induced [Ca^2+^]_i_ increase. Pretreatment of cardiomyocytes with bafilomycin A1 completely blocked the ISO-induced [Ca^2+^]_i_ increase (Fig 1C), showing that the ISO–induced sustained Ca^2+^ signal required an initial Ca^2+^ mobilization from the acidic Ca^2+^ stores.

To corroborate above observations, we tested the effect of NAADP-AM, a cell-permeable NAADP analog [34], on Ca^2+^ signaling. NAADP-AM (50 nM) induced a sustained Ca^2+^ signal in the presence, but not in the absence, of extracellular Ca^2+^ (Fig 1D). Furthermore, pretreatment of cells with 8-Br-cADPR abolished the NAADP-AM-induced sustained Ca^2+^ signal, leaving the initial Ca^2+^ transient intact (Fig 1E). Taken together, these results suggest that the ISO elicits a sequential Ca^2+^ release from acidic Ca^2+^ stores and sarcoplasmic reticulum (SR) Ca^2+^ stores by NAADP and cADPR, respectively.

To identify the Ca^2+^ influx pathway for the ISO–induced sustained Ca^2+^ signal, we studied the contribution of store-operated Ca^2+^ entry (SOCE). We used deaza-cADPR, a cell-permeable cADPR analog, to deplete Ca^2+^ from SR. Cells were incubated with 100 nM deaza-cADPR for 30 seconds in Ca^2+^-free medium and then Ca^2+^ added to the bath perfusion solution to assess the presence of SOCE, as described earlier [35]. Depletion of Ca^2+^ stores by deaza-cADPR induced Ca^2+^ entry in the presence of extracellular Ca^2+^ (Fig 1F), indicating that the sustained Ca^2+^ signal is mediated by SOCE following Ca^2+^ mobilization by cADPR. In keeping with this finding, ISO-induced sustained Ca^2+^ signals were reduced by the siRNA-mediated knockdown of Stim1 (Fig 1G).

**Fig 2:**
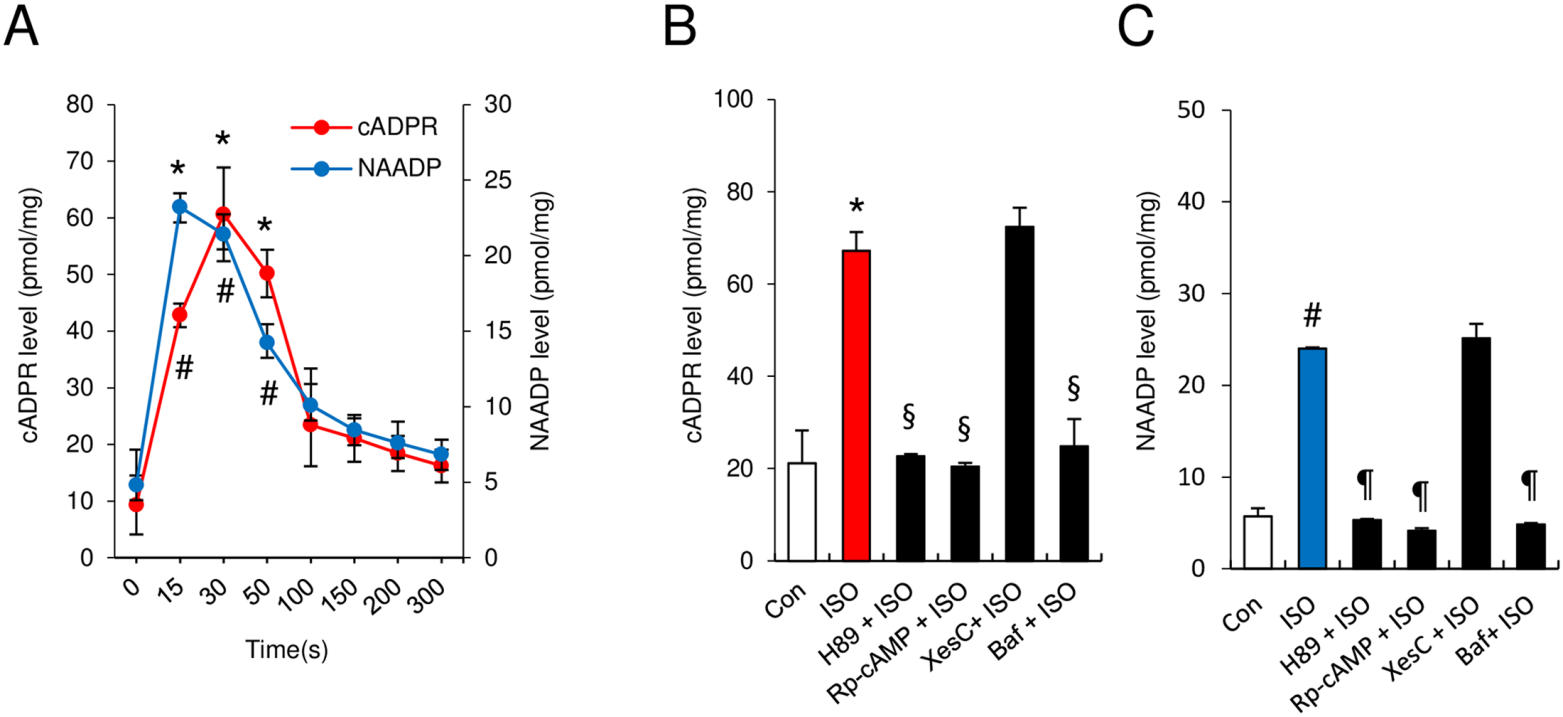
ISO-stimulated cADPR and NAADP production in cardiomyocytes. **(A)** Time course of cADPR and NAADP production following ISO treatment. **(B and C)** Differential effects of Ca^2+^ second messenger inhibitors on ISO-induced cADPR and NAADP formation. *, P<0.05 versus control (Con) cADPR level. #, P<0.05 versus control (Con) NAADP level. §, P<0.05 versus ISO-induced cADPR level. ¶, P<0.05 versus ISO-induced NAADP level. Values are the mean ± SEM of three independent experiments.

The results shown in Fig 1 suggest that NAADP and cADPR are sequentially generated in ISO-treated cardiomyocytes. To test this further, we determined the kinetics of production of these Ca^2+^-mobilizing messengers in response to ISO. Treatment of cardiomyocytes with ISO increased NAADP and cADPR production in a time-dependent manner, with peaks at about 15 and 30 s, respectively (Fig 2A). As predicted, ISO-mediated production of NAADP and cADPR was abolished in cells treated with H89 or Rp-cAMP (Fig 2B and 2C). These results indicate that the rise in cAMP level ([cAMP]) is a prerequisite for the ISO–induced production of the Ca^2+^-mobilizing second messengers, and support earlier findings that cAMP is specifically responsible for NAADP generation [24,36,37].

Bafilomycin blocked the ISO-induced formation of NAADP and cADPR to the control basal level (Fig 2B and 2C) indicating that NAADP is formed in acidic organelles and that its formation precedes the cADPR formation. This is consistent with our biochemical data also showing that formation of NAADP precedes that of cADPR (Fig 2A). Thus, NAADP appears to be the trigger for the formation of cADPR. In agreement with the lack of effect of XesC on the ISO-induced Ca^2+^ signal (Fig 1B and 1C), XesC failed to inhibit ISO-induced cADPR and NAADP production (Fig 2B and 2C). Thus, ISO stimulates the production of NAADP that in turn leads to elevation of cADPR, and these effects are independent of IP_3_-mediated Ca^2+^ release.

### CD38 Mediates ISO-induced cADPR but not NAADP Production in Cardiomyocytes

**Fig 3:**
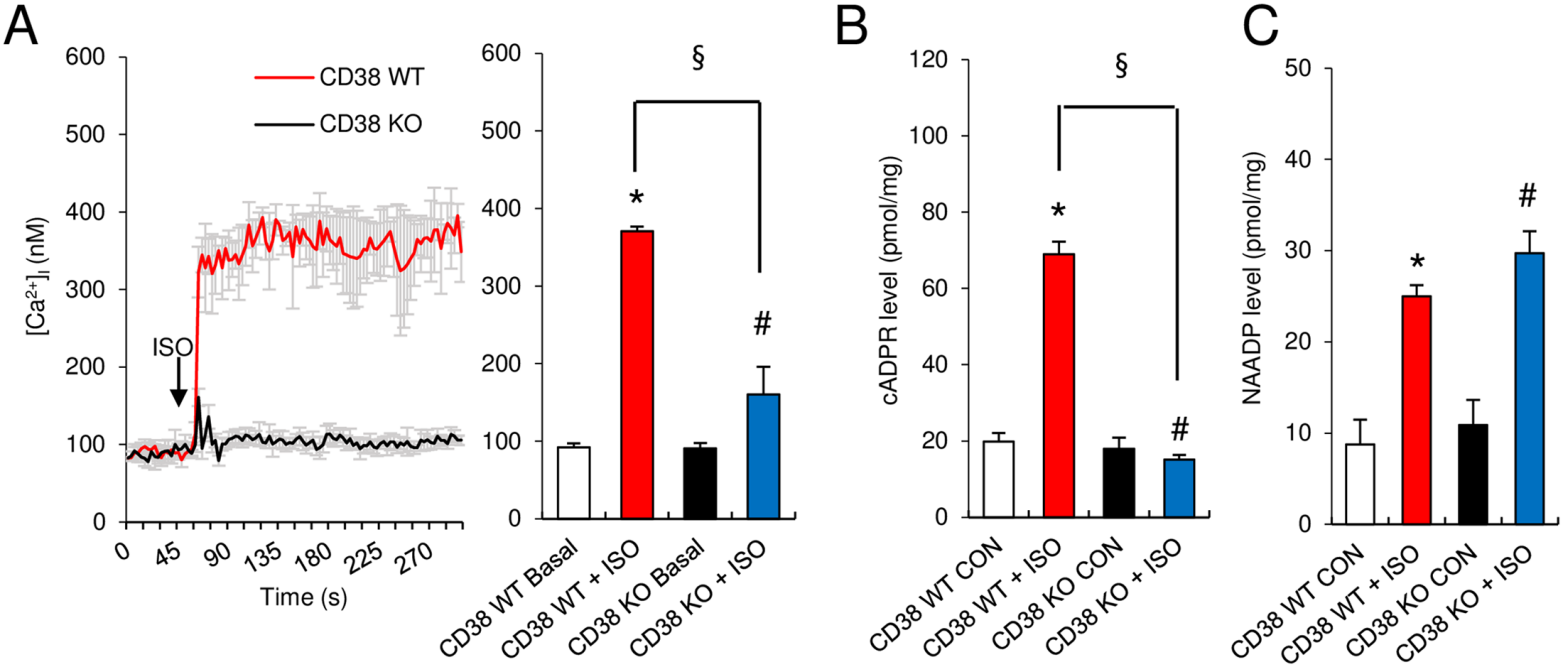
ISO-stimulated Ca^2+^ increase and cADPR and NAADP production in wild-type (WT) and CD38 knock-out (KO) mice. **(A)** Representative tracings of the Ca^2+^ response to ISO in cardiomyocytes obtained from WT and CD38 KO mice. (right panel) A direct comparison of the mean [Ca^2+^]_i_ during sustained increases in [Ca^2+^]_i._ The data shown are analyzed at 150 s. **(B)** ISO-stimulated cADPR production in WT and CD38 KO mice. **(C)** ISO-stimulated NAADP production in WT and CD38 KO mice. *, *P*< 0.05 versus WT controls. #, *P* < 0.05 versus CD38 KO controls. §, P<0.05 versus WT + ISO. Values are the mean ± SEM of three independent experiments.

Based on the above finding that the ISO-mediated increase in [Ca^2+^]_i_ is associated with NAADP/cADPR production, we investigated the role of CD38 in the ISO-mediated Ca^2+^ signaling in cardiomyocytes. ISO-induced Ca^2+^ signals were compared in cardiomyocytes isolated from CD38 KO and WT mice. The ISO-induced sustained, but not the initial transient, increase in [Ca^2+^]_i_ was abolished in ISO-treated cardiomyocytes from CD38 KO mice (Fig 3A). In keeping with this finding, ISO-induced cADPR production was abrogated in cardiomyocytes from CD38 KO mice (Fig 3B). By contrast, ISO-induced NAADP production was not reduced in cardiomyocytes from CD38 KO mice (Fig 3C). These results indicate that CD38 is responsible for cADPR production, but not NAADP production, in ISO-treated cardiomyocytes.

**Fig 4:**
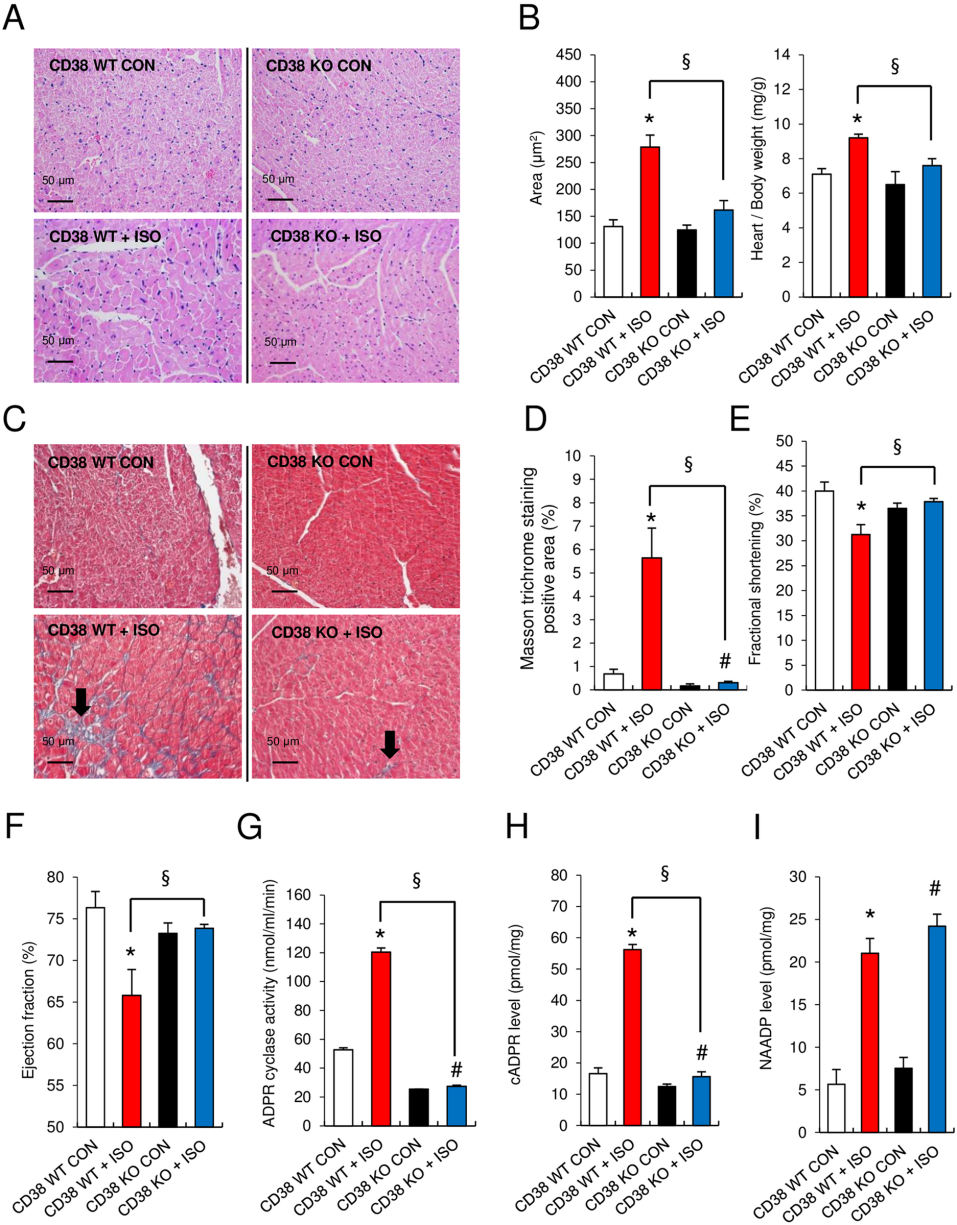
CD38 KO mice are protected from the ISO-mediated cardiac hypertrophic response. **(A)** Histological analysis of hematoxylin/eosin stained heart sections from WT and CD38 KO mice following vehicle/ISO infusion. **(B)** The mean cross-sectional areas and heart weight/body weight (HW/BW) ratios of WT and CD38 KO mice were calculated following vehicle/ISO infusion. **(C and D)** Masson’s trichrome staining reveals substantial ventricular interstitial fibrosis in WT hearts compared to CD38 KO hearts. **(E and F)** Echocardiography assessment of fractional shortening (FS) and ejection fraction (EF) following vehicle/ISO infusion in WT and CD38 KO mice. **(G-I)** ADPR cyclase activity and cADPR and NAADP formation in WT and CD38 KO mice following vehicle/ISO infusion. *, P<0.05 versus WT. #, P<0.05 versus CD38 KO. §, P<0.05 versus WT + ISO. Values are the mean ± SEM of three independent experiments.

To confirm the above finding that ISO-induced sustained increase in [Ca^2+^]_i_ requires CD38 (Fig 3), short hairpin RNA (shRNA) directed against CD38 was used to down-regulate its gene expression. Treatment of cardiomyocytes with lentiviral transduction particles containing CD38 shRNAs overnight reduced the CD38 protein level by ~90%, as assessed by immunoblot analyses (S1A Fig). Down-regulation of CD38 abolished the ISO-induced [Ca^2+^]_i_ increase observed in control cells treated with a scrambled shRNA, indicating that CD38 is necessary for ISO-mediated Ca^2+^ signaling (S1B Fig). As predicted, shRNA-mediated knockdown of CD38 also abolished the ISO-induced cADPR formation (S1C Fig). Consistent with our result that CD38 was not involved in NAADP formation (Figs 3C and 4G), shRNA-mediated knockdown of CD38 did not affect ISO-induced NAADP formation (S1D Fig). Taken together, these results show that the ISO-mediated increase in [Ca^2+^]_i_ and cADPR levels is dependent on CD38.

### Loss of CD38 Markedly Reduces the Hypertrophic Response and Preserves Cardiac Function Following Chronic ISO Infusion

The requirement of CD38 for ISO-induced sustained increase in [Ca^2+^]_i_ suggests that CD38 may also be necessary for ISO-induced hypertrophy. To test this directly, WT and CD38 KO mice were subjected to chronic infusion of ISO (Fig 4). Infusion of ISO (10 mg/kg per day) for 1 week induced advanced hypertrophy in WT mice, but not in CD38 KO mice (Fig 4A and 4B). After ISO infusion, the cell enlargement in WT mice was significantly greater than that in CD38 KO mice (Fig 4B). The cross-sectional area of cardiomyocytes increased by 113% in CD38 WT mice, but by only 30% in CD38 KO mice. The heart weight/body weight ratios (HW/BW) also increased significantly in WT mice, but not in CD38 KO mice after ISO infusion. (Fig 4B). Histological analysis revealed a significant increase in cardiac fibrosis in ISO-infused WT mice, but not in CD38 KO mice (Fig 4C and 4D). Consistent with these findings, the levels of hypertrophic and fibrosis markers (fibronectin and TGF-β1) were elevated in CD38 WT, but not CD38 KO, 7 days following ISO-treatment (S3 Fig).

Consistent with the hypertrophic and fibrotic responses to ISO in WT mice, chronic infusion of WT mice with ISO also led to a significant reduction of fractional shortening and ejection fraction, indicating an impairment of cardiac function (Fig 4E and 4F, Table 1). By contrast, cardiac function was preserved in CD38 KO mice following ISO infusion (Fig 4E and 4F, Table 1). ARC activity and cADPR levels were also not increased in ISO-infused CD38 KO mice, while NAADP levels did increase in CD38 KO mice in response to ISO (Fig 4G–4I). Collectively, these results indicate that deletion of CD38 reduces the hypertrophic response to ISO infusion and preserves cardiac function, and that cADPR-mediated Ca^2+^ signaling plays a critical role in ISO-induced cardiac hypertrophy and fibrosis.

**Table 1:** Echocardiographic assessment of CD38 WT and CD38KO mice at baseline.

**Genotype**	**CD38 WT (N = 8)**	**CD38 KO (N = 8)**
**Diastole**		
LVEDd (mm)	3.9 ± 0.22	4.1 ± 0.4
IVDT (mm)	0.55 ± 0.05	0.56 ± 0.06
PWT (mm)	0.52 ± 0.07	0.53 ± 0.06
**Systole**		
LVESd (mm)	2.5 ± 0.18	2.6 ± 0.27
IVST (mm)	0.78 ± 0.06	0.8 ± 0.07
PWT (mm)	0.8 ± 0.07	0.78 ± 0.06
EF (%)	73.2 ± 1.54	70.4 ± 1.7
FS (%)	36.7 ± 1.2	34.6 ± 1.3

Values are means ± SE

Left ventricular end-diastolic diameter (LVEDd). isovolumetric diastolic time (IVDT). Left ventricle posterior wall thickness (PWT). Ejection Fraction (EF), Fractional shortening (FS), Left ventricular mass (LV mass).

## Discussion

In the present study we demonstrate that stimulation of β-AR in cardiomyocytes by ISO induces a sustained [Ca^2+^]_i_ increase via the production of NAADP and cADPR by an NAADP-synthesizing enzyme and CD38, respectively. In WT mice, ISO induced myocardial hypertrophy, interstitial fibrosis, and cardiac dysfunction, whereas in CD38 KO mice, ISO failed to induce hypertrophy and cardiac function was preserved. Moreover, inhibition of CD38 activity ameliorated ISO-induced cardiac hypertrophy and fibrosis *in vivo*. Thus, an NAADP synthesizing enzyme and CD38 play a crucial role in ISO-induced remodeling of cardiac morphology and function.

### ISO-induced Ca^2+^ signaling via NAADP and cADPR

Sustained β-adrenergic stimulation has been implicated in the development of cardiac hypertrophy and heart failure. Indeed, altered signaling via the G-protein-coupled β-ARs has been implicated in heart failure in animal models and humans [6–8, 10]. ISO binds both β1 and β2 receptors, but only β1-receptor-mediated signaling has been shown to cause cardiac hypertrophy [38]. Although changes in Ca^2+^ metabolism have been suspected, the precise cellular mechanism for β-AR-induced development of cardiac hypertrophy is still not clear. NE released from sympathetic nerves activates cardiac β-AR and increases contractility via elevation of [Ca^2+^]_i_ [39]. Higashida et al. demonstrated that cADPR synthesis is involved in the up-regulation of cardiac function by sympathetic stimulation [17]. Injection of cADPR into the cytosol of cardiomyocytes induced Ca^2+^ sparks and potentiated the [Ca^2+^]_i_ increase by activating the ryanodine receptor and/or by increasing SR Ca^2+^ content [40,41]. Recently, Nebel et al. demonstrated a pathological role for NAADP in the generation of cellular arrhythmia induced by strong β-adrenergic stimulation [41]. These findings suggest that Ca^2+^ signaling mediated by cADPR/NAADP plays a role in pathophysiological events that occur in response to sympathetic stimulation of the heart.

In our study, ISO induced a sustained [Ca^2+^]_i_ rise that was abolished by 8-Br-cADPR, an antagonist of cADPR. Bafilomycin A1, a vacuolar H^+^ ATPase inhibitor that depletes acidic Ca^2+^ stores [24], also abolished the ISO-induced [Ca^2+^]_i_ rise and eliminated the production of both cADPR and NAADP. These results indicate that the ISO-induced sustained [Ca^2+^]_i_ increase occurs through a combination of Ca^2+^ release from non-SR and SR Ca^2+^ stores by NAADP and cADPR, respectively. The results obtained using bafilomycin A1 and 8-Br-cADPR, and biochemical measurements of cADPR and NAADP suggest the presence of a sequential activation rather a simultaneous one. Our data support a mechanism whereby Ca^2+^ release elicited first by NAADP mediates the formation of cADPR that then causes Ca^2+^ release from SR.

**Fig 5:**
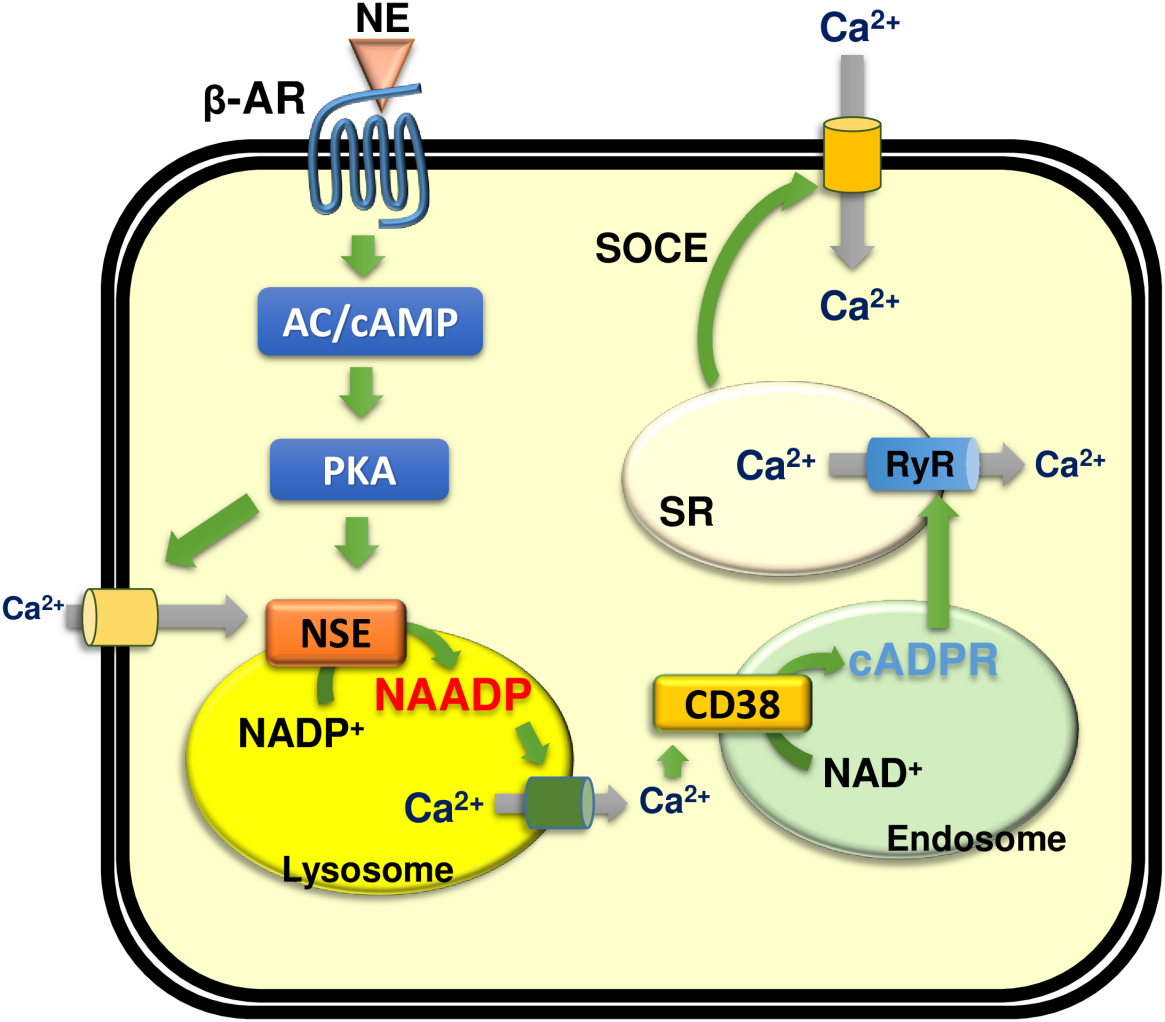
A schematic model showing signaling pathways underlying β-AR-mediated NAADP-synthesizing enzyme and CD38 activation. Binding of ISO to β-AR stimulates AC, thus activating PKA. PKA induces Ca^2+^ influx that leads to activation of an NAADP-synthesizing enzyme (NSE) to produce NAADP. NAADP-mediated Ca^2+^ mobilization from the acidic Ca^2+^ stores results in activation of CD38. cADPR produced by CD38 in the endocytic vesicles induces Ca^2+^ release from SR Ca^2+^ stores. cADPR-mediated Ca^2+^ release induces SOCE, resulting in a sustained Ca^2+^ signal. NE: norepinephrine; SR: sarcoplasmic reticulum; NSE: NAADP-synthesizing enzyme; AC, adenylyl cyclase; PKA, protein kinase A; SOCE, store-operated Ca^2+^ entry.

β-AR is normally coupled to adenylyl cyclase via Gs and elevates [cAMP] in cardiomyocytes. Our finding that PKA inhibitors block the ISO-induced rise in [Ca^2+^]_i_ and production of NAADP and cADPR indicates that, upon stimulation of β-AR, CD38 and an NAADP-synthesizing enzyme are activated by a cAMP-dependent mechanism. At present, how cAMP causes activation of these enzymes is not clear. The observation that NAADP-AM, a cell-permeable NAADP analog, induces a sustained Ca^2+^ signal only in the presence of extracellular Ca^2+^ suggests that ISO-induced Ca^2+^ signal is initiated by Ca^2+^ influx which is dependent on PKA (Fig 1). Therefore, cAMP may be the trigger that initiates the Ca^2+^ influx, leading to the activation of an NAADP synthesizing enzyme. NAADP-induced Ca^2+^ release from acidic stores would then activate CD38 to generate cADPR and cause SR Ca^2+^ depletion and induction of SOCE, resulting in a sustained Ca^2+^ signal (Fig 5). Concerning the effect of CD38 on the expression of β-AR, β1-AR and β2-AR levels of CD38 WT and CD38 KO were similar (S2 Fig). The level of β2-AR, but not β1-AR, was decreased by ISO treatment in both groups (S2 Fig), consistent with the data of Dangel et al [42] who showed that ISO-treated cardiac cells displayed changes of β1:β2 ratio, due to the decreased β2 receptor in ISO treated cells. Thus, β-AR expression was not affected by CD38.

### Role of a non-CD38, NAADP-synthesizing enzyme in ISO-induced Ca^2+^ signaling

CD38 is an ARC that is capable of producing both cADPR and NAADP. Our data obtained with CD38 KO mice and CD38 knockdown (Figs 3D, 4I and S1D) show that ISO–induced NAADP production is not dependent on CD38. Therefore, multiple enzymes probably exist for the generation of cADPR/NAADP in the heart, and cardiomyocytes very likely express an NAADP-synthesizing enzyme that is regulated in a cAMP-dependent manner. This is supported by the observation that extracts prepared from the tissues of CD38 KO mice, including the heart, were incapable of synthesizing NAADP by the base-exchange reaction *in vitro*, and that the *in vivo* cellular NAADP content was not reduced by the absence of CD38 [26]. Therefore, neither CD38 nor the base-exchange reaction may be necessary for the *in vivo* generation of NAADP. Consistent with this finding, our data (Figs 3D, 4I and S1D) clearly demonstrate that CD38 is not responsible for NAADP generation in ISO–stimulated cardiomyocytes. It will be interesting to determine how each ARC enzyme is linked to a specific downstream signaling pathway in physiological and pathological states.

### Role of ARC in ISO-induced cardiac dysfunction

An important finding of this study is that CD38 KO mice are protected from pathological hypertrophy induced by chronic ISO infusion. ISO infusion increased ARC activity and cADPR production in WT mice, resulting in ventricular fibrosis and hypertrophy and impaired cardiac function. ISO did not produce such detrimental cardiac effects in CD38 KO mice. In line with these findings, ISO-mediated [Ca^2+^]_i_ increase and cADPR production were abolished in cardiomyocytes obtained from CD38 KO mice or in cardiomyocytes in which CD38 was knocked down with shRNA. Thus, CD38 is responsible for eliciting the ISO-induced [Ca^2+^]_i_ response and the synthesis of cADPR.

We previously demonstrated that inhibition of a non-CD38 ARC by a small molecule inhibitor called 2DAB blocked Ang II-induced sustained [Ca^2+^]_i_ increase and production of cADPR in adult rat cardiomyocytes. Moreover, 2DAB attenuated Ang II-induced hypertrophic responses as indicated by the decrease in NFAT nuclear translocation, TGF-1β protein expression, and [^3^H]-leucine incorporation [27]. Consistent with these findings, 2DAB was capable of preventing the development of cardiac hypertrophy in rat renovascular hypertension model, a renin angiotensin-dependent two-kidney one-clip model of hypertension [27]. Taken together, our findings suggest that CD38 and/or non-CD38 ARCs in the myocardium could be targets for the treatment of cardiac hypertrophy. Specific intervention should be possible if the exact underlying cellular mechanisms of pathogenesis of the cardiac hypertrophy are determined.

### Comparison with AngII-induced Ca^2+^ signaling

In contrast to the dependency of ISO-mediated Ca^2+^ signaling on CD38 and NAADP-synthesizing enzyme, AngII was found to induce cardiac hypertrophic remodeling and increase cADPR production in a CD38-independent manner [23]. The difference in the modes of Ca^2+^ signaling elicited by ISO and AngII can be attributed to activation of different GPCRs. Several lines of evidence indicate that stimulation of AngII receptor activates ARC to produce cADPR in cardiomyocytes [39]. Our recent study show that AngII activates an ARC other than CD38 to induce a sustained rise in [Ca^2+^]_i_ and cardiac hypertrophy [27]. Inhibition of ARC with 2DAB significantly attenuated AngII-induced cardiac hypertrophic responses in an AngII-dependent model of hypertension [27]. Furthermore, 2DAB significantly inhibited AngII-mediated cADPR formation and hypertrophic responses in vitro. Therefore, a non-CD38 ARC is likely to be coupled to AngII receptor. Thus, β-AR and AngII receptors recruit different effectors (CD38 or non-CD38 ARC) to produce a common product, cADPR, to produce pathological hypertrophy.

Another difference between the two systems is IP_3_-dependency; AngII receptor coupled to IP_3_ production [23], but β-AR is not (Fig 1B). β-AR-induced Ca^2+^ signals are mediated by NAADP and cADPR, while AngII receptor-coupled Ca^2+^ signals are mediated by IP_3_ and cADPR [23]. These differences highlight the complexity of intracellular Ca^2+^ signaling, but ensure that each signaling pathway is unique [Fig 1 and ref [23], see scheme in Fig 5]. Although the mechanisms by which ISO and AngII modulate early Ca^2+^ signals are different, the distal targets of elevated [Ca^2+^]_i_ including CAMKII and calcineurin that regulate the cardiac gene expression and cause the development of cardiac hypertrophy are probably similar (see scheme in Fig 5).

In conclusion, our data show that chronic stimulation of β-adrenergic signaling with ISO activates an NAADP synthesizing enzyme and CD38 to increase [Ca^2+^]_i_ by elevating NAADP and cADPR levels, respectively. The sustained increase in [Ca^2+^]_i_ induced by ISO leads to cardiac dysfunction and cardiac hypertrophy in CD38 WT mice but not in CD38 KO mice. The recognition of the role of CD38 and other ARCs in maladaptive cardiac hypertrophy may help in the development of new therapeutic strategies to prevent hypertrophic heart diseases.

## Supporting Information

S1 Fig ISO-mediated Ca^2+^ increase and cADPR/NAADP production were abolished by downregulation of CD38 gene expression with shRNA. (A) Representative immunoblots with summary quantifications of CD38 protein expression in cardiomyocytes after infection with lentiviral particles expressing scrambled or CD38-specific short hairpin (shRNA). (B) Representative tracings of the Ca^2+^ response to ISO in cardiomyocytes infected with scrambled or CD38 shRNA. (C) ISO-induced cADPR production in cardiomyocytes after infection with scrambled or CD38 shRNA. *, *P*< 0.01 versus scrambled shRNA control. #, *P* < 0.01 versus scrambled shRNA+ ISO. Values are the mean ± SEM of three independent experiments.
(PPTX)

S2 Fig Expression of β1-adrenalnergic receptor (AR) and β2-AR in ISO-treated CD38 wild type (WT) and CD38 knockout (KO) mice heart. Heart were isolated and analyzed for protein expression before (Con) and following 7 days of ISO infusion (ISO).
(PPTX)

S3 Fig Expression of fibrogenetic factors in ISO-treated CD38 wild type (WT) and CD38 knockout (KO) mice heart. (A) mRNA level of fibronectin and TGFβ1 (B) protein expression level of fibronectin and TGFβ1. Heart were isolated and analyzed for protein expression before (Con) and following 7 days of ISO infusion (ISO).
(PPTX)

S1 Table Primers for detection of target genes. (PPTX)
