# Supplementary material for: Nicotinic Acid Adenine Dinucleotide Phosphate (NAADP) and Cyclic ADP-Ribose (cADPR) Mediate Ca2+ Signaling in Cardiac Hypertrophy Induced by β-Adrenergic Stimulation
Source: PLoS One. 2016 Mar 9;11(3):e0149125. doi: 10.1371/journal.pone.0149125 (PMC4784992; doi:10.1371/journal.pone.0149125)
Supplement: S1 Fig — (A) Representative immunoblots with summary quantifications of CD38 protein expression in cardiomyocytes after infection with lentiviral particles expressing scrambled or CD38-specific short hairpin (shRNA). (B) Representative tracings of the Ca2+ response to ISO in cardiomyocytes infected with scrambled or CD38 shRNA. (C) ISO-induced cADPR production in cardiomyocytes after infection with scrambled or CD38 shRNA. *, P< 0.01 versus scrambled shRNA control. #, P < 0.01 versus scrambled shRNA+ ISO. Values are the mean ± SEM of three independent experiments. (PPTX) [file pone.0149125.s001.pptx]

## Slide 1
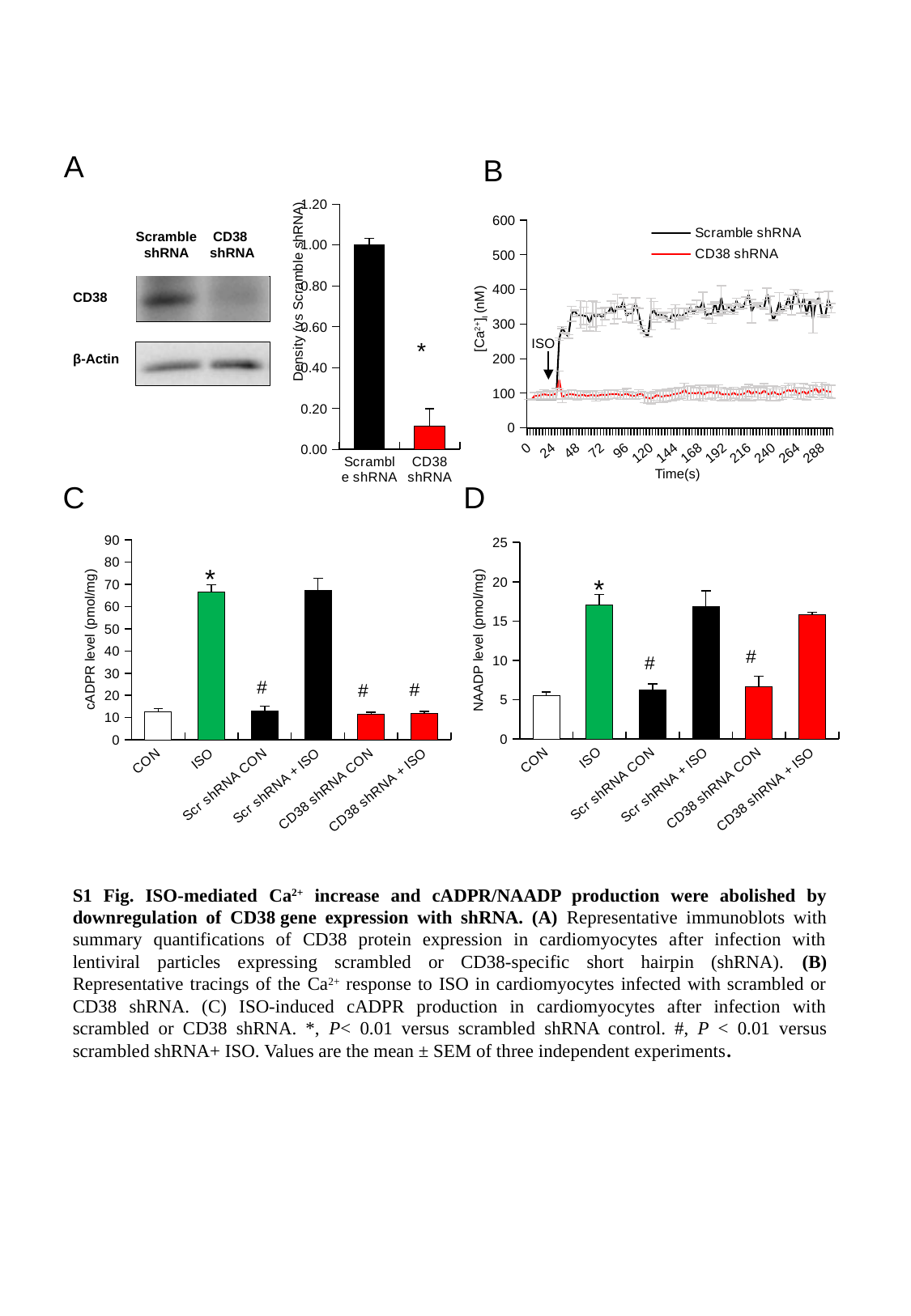

A
B
### Chart
| Category | |
|---|---|
| Scramble shRNA | 1.0 |
| CD38 shRNA | 0.1139 |
### Chart
| Category | Scramble shRNA | CD38 shRNA |
|---|---|---|
| 0 | 82.5 | 82.5 |
| 3 | 82.50005916667325 | 82.5 |
| 6 | 82.58877959319773 | 92.63875 |
| 9 | 82.41127957347551 | 92.55 |
| 12 | 82.41127957347551 | 95.02287959319773 |
| 15 | 82.41127957347551 | 97.3698 |
| 18 | 82.50005916667322 | 95.67150000000001 |
| 21 | 82.58877959319773 | 94.57131651651652 |
| 24 | 82.5 | 96.5184 |
| 27 | 82.5 | 98.85187959319774 |
| 30 | 256.4256521301 | 138.7228591556965 |
| 33 | 289.6935485876379 | 90.04393729965004 |
| 36 | 274.091047139187 | 93.40636909760359 |
| 39 | 263.9343114647655 | 97.45801569258975 |
| 42 | 329.10993700467384 | 97.16752478774748 |
| 45 | 339.4854014971255 | 96.28661455863684 |
| 48 | 324.65486159526847 | 93.91698267444765 |
| 51 | 327.8723745652875 | 93.86046763853324 |
| 54 | 323.832574981247 | 95.88080362050644 |
| 57 | 322.7736572423665 | 91.80054186643633 |
| 60 | 305.3471904979445 | 94.18072500352353 |
| 63 | 328.7052605177845 | 95.14488732747168 |
| 66 | 320.96662716100855 | 92.95068623239199 |
| 69 | 327.869005295478 | 93.72906869822629 |
| 72 | 320.1588184682132 | 96.45675064634094 |
| 75 | 330.078414361809 | 94.8112399994985 |
| 78 | 332.825876884743 | 96.2703300737236 |
| 81 | 348.74406021268203 | 97.83073002422148 |
| 84 | 330.604505598727 | 97.0220428765509 |
| 87 | 350.635745600291 | 98.2404295051354 |
| 90 | 344.803618122632 | 93.96815304865825 |
| 93 | 364.9832531993585 | 96.06598728814922 |
| 96 | 324.60701491895895 | 98.70662079316966 |
| 99 | 330.3975781034065 | 94.89439022089928 |
| 102 | 329.39893603572955 | 92.61512358542346 |
| 105 | 361.702352444006 | 92.40657639410637 |
| 108 | 327.9346067259215 | 97.81113083837778 |
| 111 | 289.6935485876379 | 98.6552855069042 |
| 114 | 274.091047139187 | 87.7555977732489 |
| 117 | 263.9343114647655 | 87.23588385186639 |
| 120 | 329.10993700467384 | 84.21628853762496 |
| 123 | 339.4854014971255 | 89.23441431099445 |
| 126 | 324.65486159526847 | 95.09048029763368 |
| 129 | 327.8723745652875 | 90.93412890834094 |
| 132 | 323.832574981247 | 90.20669239430254 |
| 135 | 322.7736572423665 | 94.09207785678375 |
| 138 | 305.3471904979445 | 92.2524212156673 |
| 141 | 328.7052605177845 | 96.74114670142743 |
| 144 | 320.96662716100855 | 97.5442174263043 |
| 147 | 327.869005295478 | 99.91583380095969 |
| 150 | 320.1588184682132 | 101.33203547539304 |
| 153 | 330.078414361809 | 110.47102684399246 |
| 156 | 332.825876884743 | 99.59082553516896 |
| 159 | 348.74406021268203 | 100.56334659001419 |
| 162 | 330.604505598727 | 100.92539932663695 |
| 165 | 350.635745600291 | 99.07778580748034 |
| 168 | 344.803618122632 | 104.33252983126457 |
| 171 | 364.9832531993585 | 95.20039707040303 |
| 174 | 324.60701491895895 | 100.60198675043927 |
| 177 | 330.3975781034065 | 103.35999574986283 |
| 180 | 329.39893603572955 | 103.47827916388692 |
| 183 | 361.702352444006 | 98.89173673717501 |
| 186 | 327.9346067259215 | 105.67988736704257 |
| 189 | 375.13317275483405 | 97.25901298802151 |
| 192 | 333.1379591543045 | 96.62749052190046 |
| 195 | 346.535304260541 | 98.58804389382837 |
| 198 | 348.2301449571255 | 94.77807096961897 |
| 201 | 331.1401023373765 | 101.58386038474927 |
| 204 | 366.869517341967 | 96.19223828858503 |
| 207 | 348.833869134203 | 96.53750544082797 |
| 210 | 345.999757438551 | 97.1660937878826 |
| 213 | 365.580253979865 | 101.43466321947444 |
| 216 | 383.931067062969 | 108.12918471483565 |
| 219 | 337.5820734845155 | 98.02580833657967 |
| 222 | 355.4677364805175 | 105.52328152217608 |
| 225 | 359.359105773528 | 101.15912731130709 |
| 228 | 347.42260399650945 | 99.19051424917794 |
| 231 | 346.26379165978403 | 107.82183196926167 |
| 234 | 386.79963459196097 | 100.21411438018697 |
| 237 | 354.3954548573255 | 95.80521059096668 |
| 240 | 311.2282155287795 | 105.58807377018292 |
| 243 | 330.014730899283 | 98.98421402603725 |
| 246 | 362.8946613088405 | 96.26515481620434 |
| 249 | 334.1867374542635 | 100.12384259891064 |
| 252 | 349.671223360202 | 105.36887246558939 |
| 255 | 375.5222144564225 | 109.44702714761826 |
| 258 | 341.47379668293695 | 105.7580776143582 |
| 261 | 391.3190467464765 | 111.74101127506465 |
| 264 | 377.723181200593 | 99.19051424917794 |
| 267 | 345.2750852863865 | 100.42726326231134 |
| 270 | 372.8685872727955 | 105.35422180483225 |
| 273 | 328.0838739799045 | 97.74523346990748 |
| 276 | 369.33792371448396 | 107.50045175508964 |
| 279 | 315.0847224959325 | 105.59775004828448 |
| 282 | 366.42550855193747 | 113.99908125274095 |
| 285 | 375.41551953390103 | 101.07247722412166 |
| 288 | 324.33624392211755 | 113.14013900212075 |
| 291 | 321.42758248985103 | 106.92156587388078 |
| 294 | 372.02778016870354 | 105.21189454260961 |
| 297 | 345.598664997914 | 104.42205988665046 |Scramble
shRNA
CD38
shRNA
Density (vs Scramble shRNA)
CD38
[Ca2+]I (nM)
ISO
*
β-Actin
Time(s)
C
D
### Chart
| Category | |
|---|---|
| CON | 12.5287 |
| ISO | 66.60929999999999 |
| Scr shRNA CON | 12.9457 |
| Scr shRNA + ISO | 67.2059 |
| CD38 shRNA CON | 11.47 |
| CD38 shRNA + ISO | 11.92 |
### Chart
| Category | |
|---|---|
| CON | 5.54332 |
| ISO | 17.0701 |
| Scr shRNA CON | 6.197759999999996 |
| Scr shRNA + ISO | 16.88159999999998 |
| CD38 shRNA CON | 6.6439999999999975 |
| CD38 shRNA + ISO | 15.792 |*
*
cADPR level (pmol/mg)
NAADP level (pmol/mg)
#
#
#
#
#
S1 Fig. ISO-mediated Ca2+ increase and cADPR/NAADP production were abolished by downregulation of CD38 gene expression with shRNA. (A) Representative immunoblots with summary quantifications of CD38 protein expression in cardiomyocytes after infection with lentiviral particles expressing scrambled or CD38-specific short hairpin (shRNA). (B) Representative tracings of the Ca2+ response to ISO in cardiomyocytes infected with scrambled or CD38 shRNA. (C) ISO-induced cADPR production in cardiomyocytes after infection with scrambled or CD38 shRNA. *, P< 0.01 versus scrambled shRNA control. #, P < 0.01 versus scrambled shRNA+ ISO. Values are the mean ± SEM of three independent experiments.
