# Supplementary material for: Nicotinic Acid Adenine Dinucleotide Phosphate (NAADP) and Cyclic ADP-Ribose (cADPR) Mediate Ca2+ Signaling in Cardiac Hypertrophy Induced by β-Adrenergic Stimulation
Source: PLoS One. 2016 Mar 9;11(3):e0149125. doi: 10.1371/journal.pone.0149125 (PMC4784992; doi:10.1371/journal.pone.0149125)
Supplement: S2 Fig — Heart were isolated and analyzed for protein expression before (Con) and following 7 days of ISO infusion (ISO). (PPTX) [file pone.0149125.s002.pptx]

## Slide 1
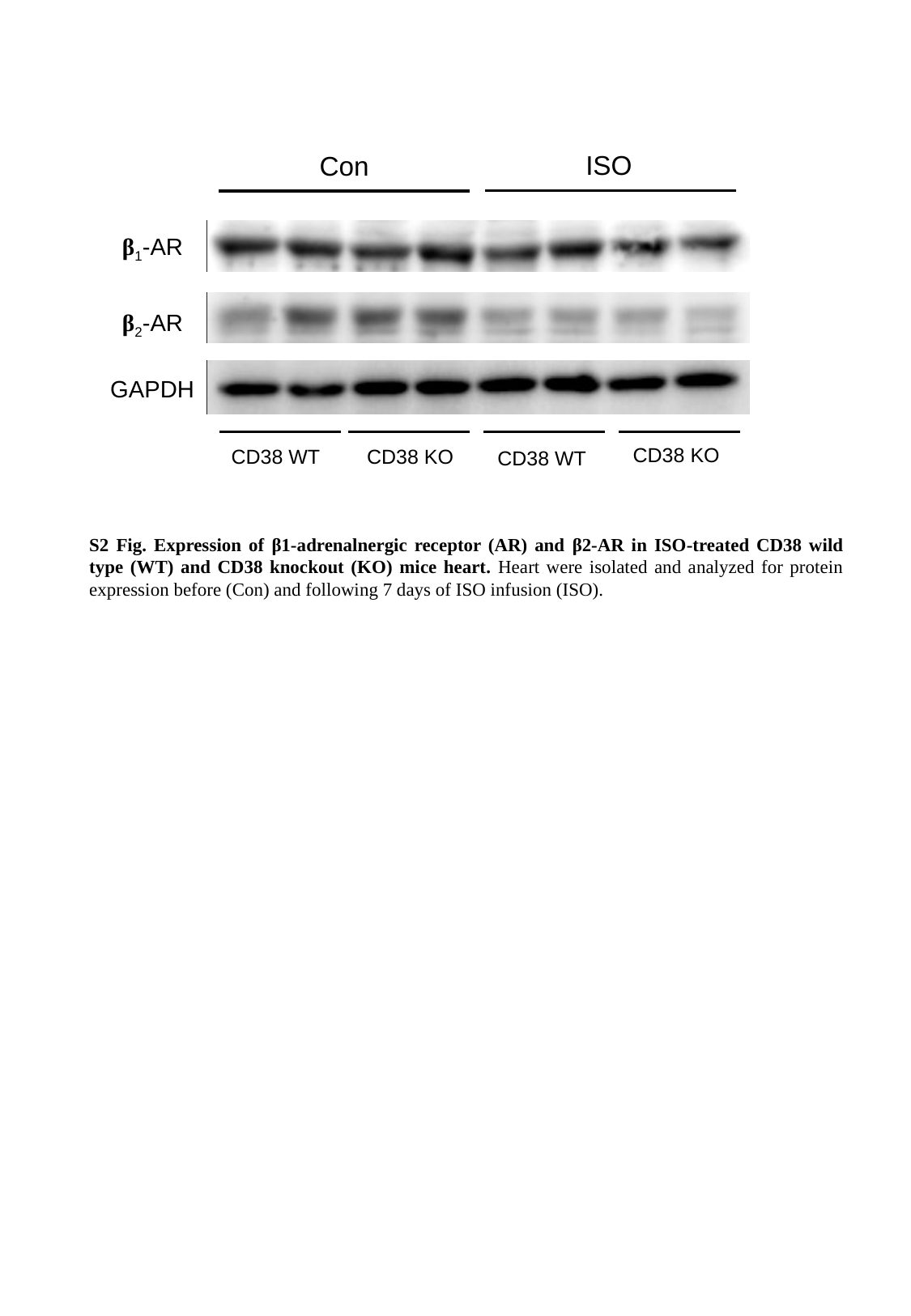

ISO
Con
β1-AR
β2-AR
GAPDH
CD38 KO
CD38 WT
CD38 KO
CD38 WT
S2 Fig. Expression of β1-adrenalnergic receptor (AR) and β2-AR in ISO-treated CD38 wild type (WT) and CD38 knockout (KO) mice heart. Heart were isolated and analyzed for protein expression before (Con) and following 7 days of ISO infusion (ISO).
