# Supplementary material for: Nicotinic Acid Adenine Dinucleotide Phosphate (NAADP) and Cyclic ADP-Ribose (cADPR) Mediate Ca2+ Signaling in Cardiac Hypertrophy Induced by β-Adrenergic Stimulation
Source: PLoS One. 2016 Mar 9;11(3):e0149125. doi: 10.1371/journal.pone.0149125 (PMC4784992; doi:10.1371/journal.pone.0149125)
Supplement: S3 Fig — (A) mRNA level of fibronectin and TGFβ1 (B) protein expression level of fibronectin and TGFβ1. Heart were isolated and analyzed for protein expression before (Con) and following 7 days of ISO infusion (ISO). (PPTX) [file pone.0149125.s003.pptx]

## Slide 1
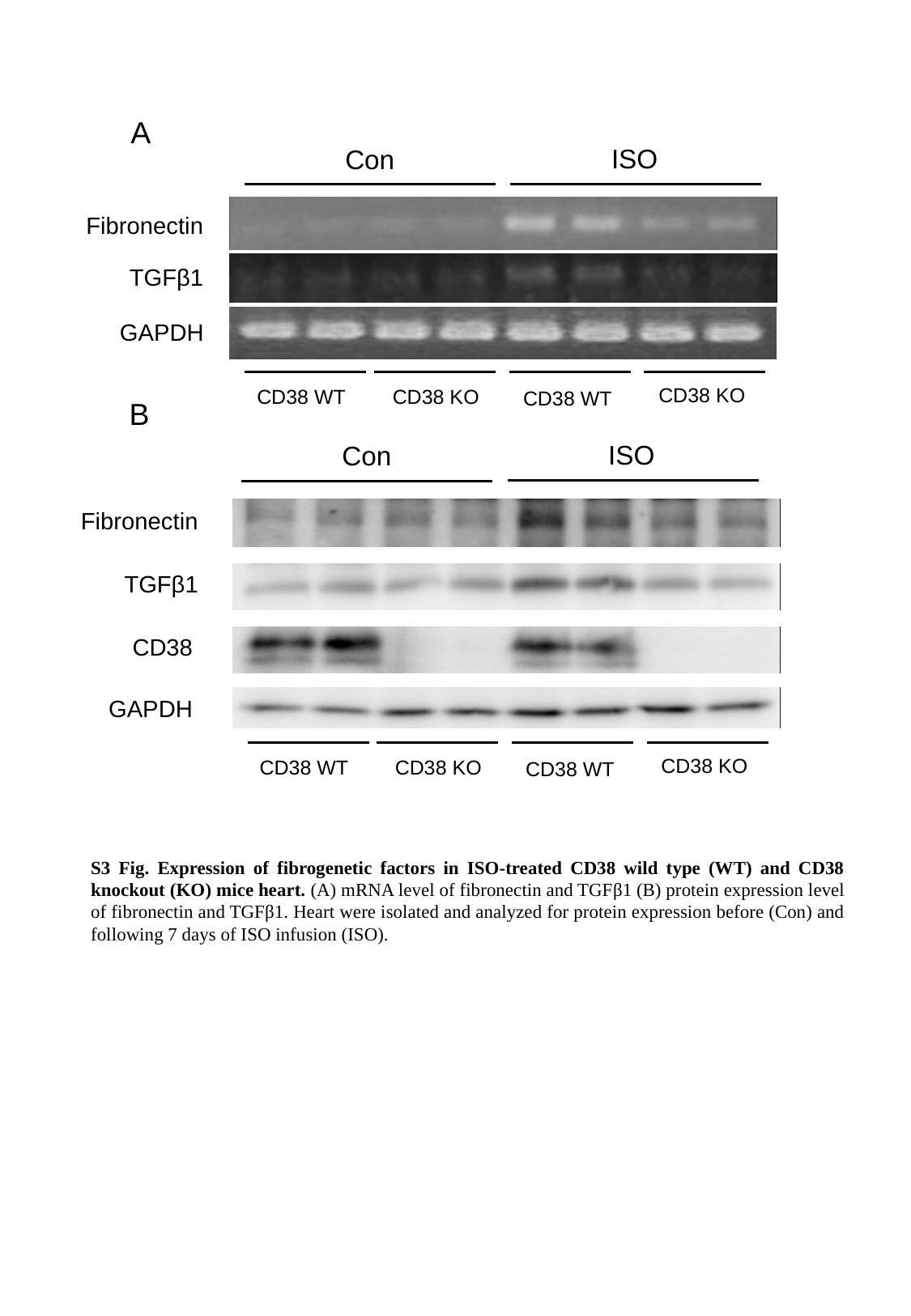

A
ISO
Con
Fibronectin
TGFβ1
GAPDH
CD38 KO
CD38 WT
CD38 KO
CD38 WT
B
ISO
Con
Fibronectin
TGFβ1
CD38
GAPDH
CD38 KO
CD38 WT
CD38 KO
CD38 WT
S3 Fig. Expression of fibrogenetic factors in ISO-treated CD38 wild type (WT) and CD38 knockout (KO) mice heart. (A) mRNA level of fibronectin and TGFβ1 (B) protein expression level of fibronectin and TGFβ1. Heart were isolated and analyzed for protein expression before (Con) and following 7 days of ISO infusion (ISO).
