# Supplementary material for: Nicotinic Acid Adenine Dinucleotide Phosphate (NAADP) and Cyclic ADP-Ribose (cADPR) Mediate Ca2+ Signaling in Cardiac Hypertrophy Induced by β-Adrenergic Stimulation
Source: PLoS One. 2016 Mar 9;11(3):e0149125. doi: 10.1371/journal.pone.0149125 (PMC4784992; doi:10.1371/journal.pone.0149125)
Supplement: S1 Table — (PPTX) [file pone.0149125.s004.pptx]

## Slide 1
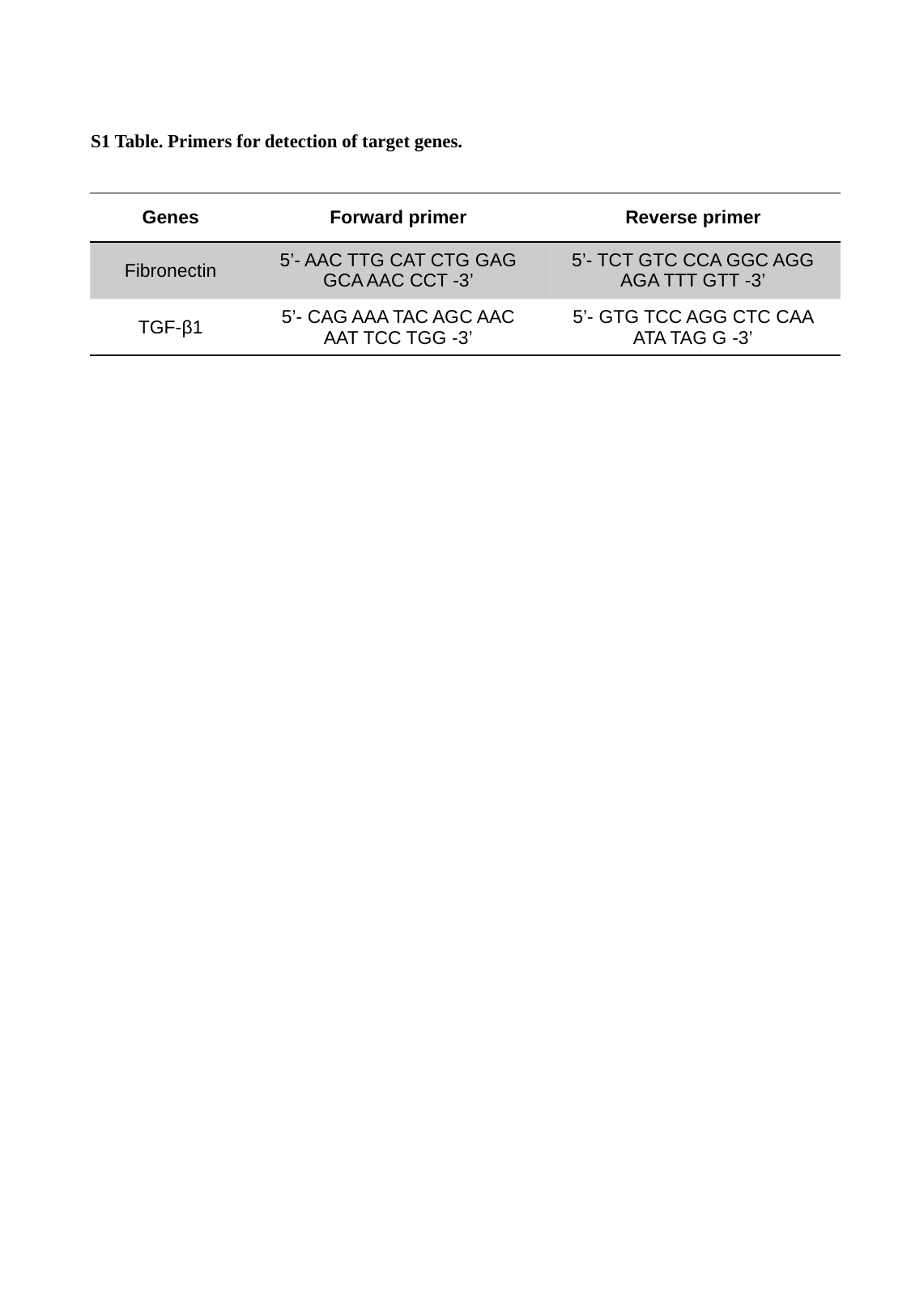

S1 Table. Primers for detection of target genes.
| Genes | Forward primer | Reverse primer |
| --- | --- | --- |
| Fibronectin | 5’- AAC TTG CAT CTG GAG GCA AAC CCT -3’ | 5’- TCT GTC CCA GGC AGG AGA TTT GTT -3’ |
| TGF-β1 | 5’- CAG AAA TAC AGC AAC AAT TCC TGG -3’ | 5’- GTG TCC AGG CTC CAA ATA TAG G -3’ |
